# Outcomes of cardiac surgery after mediastinal radiation therapy: A single‐center experience

**DOI:** 10.1111/jocs.14427

**Published:** 2020-01-23

**Authors:** Onur B. Dolmaci, Emile S. Farag, S. Matthijs Boekholdt, Wim J. P. van Boven, Abdullah Kaya

**Affiliations:** ^1^ Department of Cardiothoracic Surgery, Amsterdam UMC University of Amsterdam Amsterdam The Netherlands; ^2^ Department of Cardiology, Amsterdam UMC University of Amsterdam Amsterdam The Netherlands

**Keywords:** cardiac surgery, mediastinal radiation therapy, radiation therapy, valve surgery

## Abstract

**Background:**

Mediastinal radiation therapy (MRT) is a widely used therapy for thoracic malignancies. This therapy has the potential to cause cardiovascular injuries, which may require surgery. The primary aim of this study is to identify the perioperative outcomes of cardiac surgery in patients with a history of MRT. Second, potential predictors of mortality and adverse events were identified.

**Methods:**

A retrospective study was conducted among 59 patients with prior MRT who underwent cardiac surgery between December 2009 and March 2015. Included surgeries consisted of procedures through median‐ and ministernotomy. Baseline, perioperative, and follow‐up data were obtained and analyzed.

**Results:**

The majority of patients had a history of breast cancer (n = 43), followed by Hodgkin lymphoma (n = 10) and non‐Hodgkin lymphoma (n = 3). Preoperative estimated mortality with the Euroscore II was 3.4%. Overall 30‐day mortality was 6.8% (n = 4), with a total in‐hospital mortality of 10.2% (n = 6). Postoperatively, nine rethoracotomies (15.3%) had to be performed. During a mean follow‐up of 53 months, an additional 10 patients (16.9%) died, of which 60% (n = 6) as a result of cancer‐related events. Cox proportional modeling showed no differences in mortality between primary malignancies (*P*  > .05).

**Conclusion:**

This study shows that cardiac surgery after mediastinal radiotherapy is associated with increased short‐ and long‐term mortality when compared to preoperative mortality risks predicted by the Euroscore II. Surgery‐related events caused all short‐term mortality cases, while malignancy‐related events were the main cause of death during the follow‐up. Mortality was higher in patients with a previous stroke and a lower estimated glomerular filtration rate.

**Abbreviations**
CABG: coronary artery bypass grafting
CAD: coronary artery disease
COPD: chronic obstructive pulmonary disease
ICU: intensive care unit
MRT: mediastinal radiotherapy
TAVI: transcatheter aortic valve implantation

## 1 INTRODUCTION

Mediastinal radiation therapy (MRT) is a widely used treatment method for thoracic malignancies, especially among patients who suffer from breast cancer or thoracic lymphomas. MRT, alongside surgical procedures and chemotherapeutic treatments, has resulted in an increase in survival in these patients.^1^, ^2^ In turn, this higher survival rate led to an increase in the number of patients with long‐term MRT‐induced cardiovascular injuries.^3^ The damage caused by MRT might occur decades after the initial treatment and is currently the most common cause of nonmalignant mortality in these patients.^4^ The pathogenesis of MRT‐induced cardiovascular diseases is initiated by radiation‐induced inflammation, which subsequently leads to the development of fibrosis and calcification.^5^, ^6^, ^7^ These histological changes can develop in all components of the heart, which could lead to a wide range of cardiovascular diseases, such as valvular stenosis or regurgitation, coronary artery disease (CAD), cardiomyopathy, pericardial disease, and conduction disorders.^4^, ^8^ Recent studies have shown a high incidence of long‐term MRT‐induced cardiovascular side effects. For instance, a study among 6039 patients treated for Hodgkin lymphoma showed that 11.6% developed long‐term MRT‐induced cardiovascular disease. CAD was the most common complication, occurring in 19% of the cases.^9^ Additionally, a recent meta‐analysis including 1.2 million participants who underwent MRT as a treatment for breast cancer, indicated an increased risk of developing CAD and cardiac mortality (relative risk 1.30 and 1.38, respectively).^10^

Previous studies have already shown worse outcomes of cardiac surgery in patients with a history of MRT compared to patients without a history of MRT.^11^, ^12^, ^13^, ^14^, ^15^, ^16^, ^17^ These studies mostly researched a subset of cardiac procedures (mostly valvular surgery). We hypothesize that previous MRT is associated with an increased risk of mortality and complications in the entire spectrum of invasive cardiac procedures. The primary aim of this study is to identify the perioperative outcomes of all invasive cardiac procedures in patients with a history of MRT in a single center in The Netherlands. Furthermore, we aim to identify potential predictors of mortality and adverse events in patients undergoing cardiac surgery after MRT.

## 2 METHODS

This retrospective study was conducted at the Amsterdam University Medical Center in The Netherlands. The electronic health records of patients who underwent cardiac surgery between December 2009 and March 2015 were screened to identify all patients with a history of MRT. Patients who underwent open chest cardiac surgery, including procedures through median sternotomy and minithoracotomy/sternotomy, were included. Minimally invasive procedures such as transcatheter procedures—including transfemoral and transapical approaches—were excluded. Approval for this study was granted by the medical ethics committee and patient consent was waived.

### 2.1 Study parameters

Preoperative patient characteristics, such as comorbidities, data on previous surgeries, laboratory findings (eg, renal and cardiac markers), complications, and echocardiographic findings (eg, left ventricle function and valve function) were obtained. The New York Heart Association classification of each individual was obtained from the health records. Information about the patients' CAD status was obtained from the most recent preoperative coronary angiography. CAD, renal insufficiency, and myocardial infarction were defined according to the definitions of the Society of Thoracic Surgeons (STS) national cardiac surgery database. Euroscore I and Euroscore II were calculated for each patient.^18^ Patients who had not been electively admitted for operation, but did require intervention or surgery on the same admission for medical reasons, were classified as urgent.

Periprocedural characteristics, such as data on the operation (eg, type of surgery, cardiopulmonary bypass duration, and aortic cross clamp duration), complications, adverse events, and admission time were collected for each individual. The majority of the patients was transferred to the referring hospital when they were fit to go; therefore, the hospital admission time as reported in this study is the time between surgery and transfer to the referring hospital.

The follow‐up of the included patients was conducted until March 2018. The referring hospitals were contacted to collect information about the postoperative period. In case of incomplete information, the patients' general practitioners were contacted. Readmissions, reoperations, other interventions, related events, and mortality were reported for the follow‐up period.

### 2.2 Statistical analysis

Continuous variables are presented as mean ± standard deviation or as median and interquartile range (IQR), according to the distribution. Continuous variables were analyzed using multivariate regression analysis. Categorical data are presented as frequencies and percentages and were compared with the *χ* ^2^ test. To compare the different outcomes of various types of cancer, the Student *t* test was employed. Longitudinal survival was estimated by Kaplan‐Meier analysis. Differences in survival curves were analyzed and nonparametrically distributed variables were compared with the logrank test. Factors influencing the patients' survival were identified with the multivariate Cox proportional hazard model. Only those variables that displayed a significant association with mortality in a univariate analyses were included for the multivariate model. *P* < .05 was considered to be significant. Statistical analysis was conducted using IBM SPSS for Windows version 25.0.

## 3 RESULTS

A total of 59 patients were eligible for inclusion. Baseline characteristics of the patients are shown in Table 1. The group consisted predominantly of women (78%) with a median age of 70 years at the moment of surgery. The majority of included patients was diagnosed with breast cancer (n = 43), followed by Hodgkin and non‐Hodgkin lymphomas (n = 10 and n = 3, respectively), lung cancer (n = 2), and one case of an extragonadal germ cell tumor located in the thorax. Twenty‐one patients (35.6%) had been treated with chemotherapy in the past. Preoperative echocardiographic findings are shown in Table 2.

**Table 1:** Baseline characteristics

Characteristic	Total (n = 59)
Age at surgery, y	70 (65‐76)
Sex (n, %)	
Female	46 (78)
Smoking (n, %)	
Never	36 (61)
Ex‐smoker	11 (18.6)
Current smoker	8 (13.6)
Body mass index (kg/m²)	26.1 (23.6‐28.3)
Comorbidity (n, %)	
Hypertension	41 (69.5)
Diabetes mellitus	15 (25.4)
COPD	8 (13.6)
Cardiovascular comorbidity (n, %)	
Atrial fibrillation	13 (22.0)
Myocardial infarction	8 (13.6)
Myocardial infarction, <90 d	7 (11.9)
TIA	4 (6.8)
Stroke	1 (1.7)
Peripheral vascular disease	2 (3.4)
Pacemaker	2 (3.4)
Coronary artery disease (number of arteries)	
0	24 (40.7)
1	11 (18.6)
2	5 (8.5)
3	19 (32.2)
NYHA classification (n, %)	
1	3 (5.1)
2	32 (54.2)
3	21 (35.6)
4	3 (5.1)
Euroscore	
I—Standard	6.07 ± 2.25
I—Logistic	5.5 (3.5‐7.9)
II	2.4 (1.4‐4.0)
Prior cardiac intervention (n, %)	
PCI	13 (22.0)
Ablation	1 (1.7)
CABG	2 (3.4)
Valve	1 (1.7)
CABG + valve	1(1.7)
Other	2 (3.4)
ASA score at surgery (0‐4)	2.89 ± 0.41
Preoperative creatinine (μmol/L)	82 (67‐92)

*Note*: Data are presented as n (%), mean ± SD, or median (interquartile range).

Abbreviations: AF, atrial fibrillation; ASA, American Society of Anesthesiologists score; CABG, coronary artery bypass grafting; COPD, chronic obstructive pulmonary disease; NYHA, New York Heart Association (functional class preoperatively); PCI, percutaneous coronary intervention; TIA, transient ischemic attack.

**Table 2:** Preoperative echocardiographic characteristics

Characteristic	Total (n = 59)
Left ventricular function (ejection fraction; n, %)	58/59^a^
Normal (>50%)	42 (71.2)
Mildly impaired (40%‐49%)	14 (23.7)
Moderately impaired (25%‐39%)	2 (3.4)
Severely impaired (<25%)	⋯
Aortic stenosis (peak gradient in mm Hg)	67.6 (56.2‐87.8)
Aortic stenosis (mean gradient in mm Hg)	37.5 (30.8‐42.8)
Aortic valve area, cm²	0.72 (0.69‐0.84)
Aortic regurgitation (grades 0‐4)	1.02 ± 1.00
Mitral regurgitation (grades 0‐4)	1.33 ± 1.23
Tricuspid regurgitation (grades 0‐4)	0.86 ± 1.07

*Note*: Data are presented as n (%), mean ± SD, or median (interquartile range).

^a^ Denominator represents number of patients for whom this information was known.

The median time interval between the last received MRT and surgery was 21 years (IQR, 8.75‐28). The cardiac surgeries that were most often performed were single‐valve repair/replacement, isolated coronary artery bypass grafting (CABG), and CABG combined with a single‐valve repair/replacement (31%, 29%, and 22%, respectively). Coronary revascularization was most often achieved by using the vena saphena magna as graft material (n = 31). The second most often employed graft was the left internal mammary artery (n = 22, 27.8%). Both the right internal mammary artery and the radial artery were used only once. Preoperative imaging of the aorta (to detect potential calcifications of the aorta as a result of MRT) was not performed in any patient; however, five patients were operated without the use of cardiopulmonary bypass (off‐pump CABG). A total of six (10.2%) patients received a concomitant procedure besides the procedures as described above. Six of the included cases were a redo sternotomy (10.2%). Another six (10.2%) patients underwent urgent surgery because of acute symptomatic CAD (n = 3), decompensated aortic valve stenosis (n = 2), and one case with native aortic valve endocarditis. Detailed information about the perioperative characteristics is shown in Table 3.

**Table 3:** Perioperative characteristics

Characteristic	Total (n = 59)
Urgent surgery (n, %)	6 (10.2)
Performed surgery (n, %)	
CABG isolated	13 (22.0)
CABG + 1 valve	17 (28.8)
CABG + >1 valve	5 (8.5)
1 Valve repair/replacement	18 (30.5)
>1 Valve repair/replacement	5 (8.5)
PAPVC correction	1 (1.7)
Aortic valve replacement (n, %)	39 (66.1)
Mitral valve (n, %)	13 (22.0)
Replacement	8 (13.6)
Repair	5 (8.5)
Tricuspid valve repair (n, %)	5 (8.5)
Number of grafts	1.49 ± 1.58
Incision type (n, %)	
Median full sternotomy	56 (94.9)
Ministernotomy (J‐sternotomy)	2 (3.4)
Minithoracotomy	1 (1.7)
ECC time, min	118 (89‐169)
Cross‐clamp time, min	85 (62‐117)

*Note*: Data are presented as n (%), mean ± SD, or median (interquartile range).

Abbreviations: CABG, coronary artery bypass grafting; ECC, extracorporal circulation time; PAPVC, partial anomalous pulmonary venous connection; TAVI, transcatheter aortic valve implantation.

## 4 POSTOPERATIVE OUTCOMES

### 4.1 Short‐term (in‐hospital) mortality

This study did not include any cases of periprocedural mortality (within 24 hours after surgery). A total of six patients (all female) died during their stay in the hospital, of which four within 30 days after surgery. Three out of four of these patients were treated for breast cancer in the past. All short‐term mortalities were cardiovascular related. Detailed information about the in‐hospital deaths can be found in Table 4.

**Table 4:** Details of in‐hospital deaths

Age, y	Sex	Euroscore II	MRT to surgery, y	NYHA	Surgical procedure	ECC time, min	Cross‐clamp time, min	Interval surgery‐death, d	Indication for rethoracotomy	Cause of death	Autopsy findings
82	F	3.60	23	3	AVR	N/A	N/A	4	Hemothorax	Refractory shock	Diffuse myocardial damage and early hepatic necrosis
82	F	2.97	⋯	2	CABG + AVR	237	135	2	⋯	MOF after DC postinfarction after graft failure	⋯
66	F	6.59	33	3	CABG,AVR,MVR	286	169	38	Hemorrhage	MOF after LVF and RVF	⋯
69	F	6.64	20	2	AVR	87	45	9	⋯	MOF after Sepsis + forward and backward failure	⋯
67	F	9.49	2	3	ASD closure, PAPVC rerouting, TVP, MVP	174	92	41	Post‐CPR + signs of Mediastinitis	MOF with signs of systemic infection	Recent left ventricular infarction, lung edema, chronic DC, paravalvular dehiscention
64	F	5.31	10	2	ASD/VSD closure, MVR, TVP	205	94	8	First LVAD placement	MOF after cardiac and distributive shock	Generalized edema, signs of (sub)acute DC, toxic myocardial damage
									Second Hematoma		

Abbreviations: ASD, atrial septum defect; AVR, aortic valve replacement; CABG, coronary artery bypass graft surgery; CPR, cardiopulmonary resuscitation; DC, cardiac decompensation; ECC, extracorporal circulation time; F, female; LVAD, left ventricular assist device; LVF, left ventricle failure; min, minutes; MOF, multiorgan failure; MRT, mediastinal radiotherapy; MVP, mitral valve plasty; MVR, mitral valve replacement; N/A, not applicable; NYHA, New York Heart Association; PAPVC, partial anomalous pulmonary venous connection; RVF, right ventricle failure; TVP, tricuspid valve plasty; VSD, ventricular septum defect.

### 4.2 Long‐term (postdischarge) mortality

**Figure 1:**
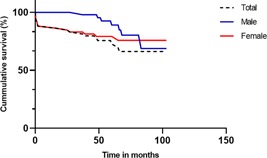
Kaplan‐Meier survival analyses of all patients. Logrank testing showed no significant differences in mortality between male and female

The mean follow‐up period was 53 months (range, 1‐103 months), resulting in 261 patient years of follow‐up. The follow‐up was less than 12 months in only seven patients, because all of them died within 12 months after surgery (including the six cases of in‐hospital death). An additional number of 10 patients (16.9%) died during follow‐up (Figure 1). Median interval between surgery and death of these patients was 42.5 months (IQR, 12.5‐61.3). Two of these patients died shortly after surgery (at 1 and 2 months). One patient died of a suspected mediastinitis and endocarditis. The cause of death of the other patient was not clarified.

Of the remaining eight patients, six died because of a malignancy, one because of a cardiac cause, and one due to an unknown cause. The patient who died as a consequence of a cardiac problem suffered from right ventricular failure after a complicated subtotal pericardiectomy, which was planned because of an underlying constrictive pericarditis, 27 months after the primary surgery.

### 4.3 Short‐term (in‐hospital) complications and reinterventions

Postoperatively, nine (15.3%) rethoracotomies had to be performed (Table 5), seven of which caused by a hemorrhage (of which two showed signs of a tamponade), one patient with an occluded graft which required a re‐CABG, and finally, a patient with signs of a mediastinitis. During admission, four patients (6.8%) required cardiopulmonary resuscitation; underlying causes were right ventricular failure, cardiac stunning with intravascular volume depletion, electromechanical dissociation, and a persistent asystole after atrial fibrillation. Twelve patients (20.3%) developed serious cardiac complications (decompensation or myocardial infarction) and six (10.2%) developed neurological complications—as reported in Table 5. New arrhythmias (mostly atrial fibrillation) were seen in 25 patients (42.4%) and 8 patients (13.6%) developed conduction disorders (eg, atrioventricular‐block) which eventually resulted in pacemaker implantation in 3 of them (5.1%). Sternal wound infections occurred in two patients (3.4%).

**Table 5:** Postoperative characteristics (before discharge)

Characteristic	Total
	(n = 59)
30‐d Mortality (n, %)	4 (6.8)
In‐hospital mortality (n, %)	6 (10.2)
Rethoracotomy (n, %)	9 (15.3)
Other reinterventions (n, %)	
PCI	6 (10.2)
Thorax drainage	4 (6.8)
Pacemaker	3 (5.1)
Cardiopulmonary resuscitation, in patients (n, %)	4 (6.8)
Complications (n, %)	58/59^a^
Cardiac	
Atrial fibrillation (new onset)	25 (42.4)
Conduction abnormalities	8 (13.6)
Cardiac decompensation	8 (13.6)
Myocardial infarction	7 (11.9)
Neurological	
Delirium	4 (6.8)
TIA	1 (1.7)
Stroke	1 (1.7)
Infectious	
Pneumonia	6 (10.2)
Urinary tract infection	3 (5.1)
Sepsis/SIRS	3 (5.1)
Sternal wound infection	2 (3.4)
Pericarditis	1 (1.7)
Renal	
Renal insufficiency	10 (16.9)
Dialysis	7 (11.9)
Creatinine, μmol/L	90 (68‐124)
GFR, mL/min/1.73 m^2^	61.5 ± 26.6
ICU duration, d	3 (2‐5)
Hospital stay length, d	8 (6‐12)
Max CK‐MB, U/L	58/59^a^
	34.6 (21.0‐76.7)

*Note*: Data are presented as n (%), mean ± SD, or median (interquartile range).

Abbreviations: AF, atrial fibrillation; CK‐MB, creatine kinase‐myoglobin; GFR, glomerular filtration rate; ICU, intensive care unit; PCI, percutaneous coronary intervention; SIRS, systemic inflammatory response syndrome; TIA, transient ischemic attack.

^a^ Denominator represents a number of patients for whom this information was known.

### 4.4 Long‐term (postdischarge) complications and reinterventions

Three patients needed a rethoracotomy after discharge. One patient required a second rethoracotomy 14 days after the initial surgery, due to an old hemorrhage with signs of tamponade. A lavage and Hickman catheter placement was required in a patient with mediastinitis (7 months after primary surgery). The third case included a patient with a planned subtotal pericardiectomy, who died shortly after surgery as mentioned above (27 months after primary surgery). Other additional interventions after discharge were one pacemaker implantation (1.7%), six coronary angiographies (10.2%), five thorax drainages (8.5%), and two percutaneous coronary interventions (3.4%). Nine patients (15.3%) had to be readmitted during follow‐up. The reasons for admission were cardiac decompensation (n = 4, 6.8%), angina pectoris (n = 2, 3.4%), and one case of thrombosis of a prosthetic valve.

## 5 IDENTIFIED RISK FACTORS

### 5.1 Mortality

To identify risk factors for mortality, Cox proportional hazard modeling was performed (Table 6). The analysis revealed a higher incidence of all‐cause mortality in patients a previous stroke (hazard ratio, 17.3; 95% CI, 1.76‐171.4; *P* = .015). A negative relationship was found between estimated glomerular filtration rate (eGFR) and all‐cause mortality (hazard ratio, 0.97; 95% CI, 0.94‐0.99; *P* = .009 per mL/min/1.73 m^2^). As expected, univariate analyses indicated that both Euroscore I and Euroscore II were predictors of mortality (hazard ratio, 1.35; 95% CI, 1.06‐1.73; *P* = .016, and hazard ratio, 1.16; 95% CI, 1.03‐1.30; *P* = .016, respectively). However, both scores underestimated the actual mortality risk in the patients included in this study. The mean Euroscore I and II estimates of mortality risk in this group were 6.1% and 3.4%, respectively, while the actual mortality was 10.2%. Age was also found to be a determinant of mortality (hazard ratio, 1.129; 95% CI, 1.003‐1.27; *P* = .044). When assessing the difference in mortality between various types of cancer, analyses showed no significant differences of mortality outcomes.

**Table 6:** Multivariate Cox proportional hazard model for survival

Characteristic	Hazard ratio (95% CI)	*P* value
eGFR	0.968 (0.94‐0.99)	.009
Previous stroke	17.3 (1.76‐171.4)	.015
Hypertension	9.8 (0.7‐46.4)	.094

Abbreviations: CI, confidence interval; eGFR, estimated glomerular filtration rate.

### 5.2 Adverse events

Patients who were diagnosed with chronic obstructive pulmonary disease (COPD) had a longer intensive care unit (ICU) stay, compared to those without COPD *(P *= .034). Furthermore, Euroscore II was associated with a longer ICU stay (*P* = .035). Further analyses looked into malignancy‐related adverse events. These results indicated that those patients with breast cancer in the past had a higher incidence of postoperative kidney failure (*P* = .001). No differences were found in postoperative outcomes between patients with left‐sided vs right‐sided breast cancer (*P* > .05). Furthermore, a history of chemotherapy was not associated with worse outcomes (*P* > .05). The time between MRT and surgery also showed no association with adverse outcomes (*P* > .05). Preoperative ejection fraction (EF) or valve‐related problems were not related to adverse events. However, selection bias could have painted a wrong image, since the majority of the patients in this study (71.2%) had a good EF (>50%) before surgery.

## 6 DISCUSSION

The primary aim of this study was to identify the perioperative outcomes of invasive cardiac procedures in patients with a history of MRT. Over the last decades, the number of patients surviving cancer has risen dramatically, at the price of long‐term MRT‐induced cardiovascular complications.^3^, ^19^ MRT inflicts radiation‐induced inflammation in the radiation field, which subsequently leads to the development of fibrosis and calcification. Depending on the cardiac structures captured in the radiation field, this could result in problems like valvular damage, accelerated CAD, and conduction disorders.^5^, ^6^, ^7^ These comorbidities can affect the perioperative outcomes of surgery later in life. Therefore, it is important to know what the outcomes of cardiac surgery are in patients with previous MRT.

Whereas previous studies have mainly focused on valvular cardiac surgery after MRT, this study took all invasive cardiac surgeries into account. The current study adds to the notion that mortality of cardiac surgery is higher for patients with a history of MRT. Surgery‐related events were the cause of death for all short‐term mortality cases (n = 6). Additionally, two patients died shortly after discharge, of which one from surgery‐related events and one as a result of an unidentified cause. Mortality during late follow‐up was mainly caused by malignancy‐related events (6/8, 75%). Furthermore, this study showed that risk calculators like the Euroscore II underestimate the risk of mortality in patients with a history of MRT (3.4% vs 10.2%).

Since three out of four patients died as a result of malignancy‐related events during late follow‐up instead of cardiac‐ or surgery‐related events, one could argue that undergoing surgery had a positive impact on these patients' health. This implicates that cardiac surgery should not be ruled out as a treatment for patients with a history of MRT. Minimally invasive approaches such as transcatheter aortic valve implantations (TAVIs) could be considered as an alternative to conventional surgery at high‐risk patients with severe aortic valve stenosis. Percutaneous approaches (eg, transfemoral and transapical) could have beneficial outcomes since they eliminate the issues caused by the late effects of MRT, such as the calcification of the aorta (problems with cannulation and clamping) and extensive mediastinal fibrosis. Dijos et al^20^ already showed promising outcomes of TAVI with low postoperative mortality and complications and good hemodynamic results. Future studies will have to compare the results of conventional surgery vs TAVI in patients with previous MRT and aortic valve stenosis.

Calculators used for predicting the risk of mortality in patients undergoing cardiac surgery are the Euroscore II and the STS risk score. Recent evidence shows that these calculators might not be as reliable in predicting the mortality of patients with a history of MRT undergoing cardiac surgery as previously expected.^14^, ^15^, ^17^ The mortality in the current study was higher than estimated by the Euroscore II (10.2% vs 3.4%), matching the results found by Ghoneim et al^15^ and Wu et al.^17^ This implicates that the Euroscore II is suboptimal in calculating the risk of mortality in patients with a history of MRT.

In‐hospital mortality rates after cardiac surgery in a general patient population without previous MRT varies between 3% and 4%.^18^, ^21^ In the current study, the in‐hospital mortality was substantially higher (10.2%). Previous studies, which compared the data of patients undergoing valvular surgery with previous MRT vs matched groups without MRT, showed higher rates of in‐hospital mortality and worse survival of patients with MRT.^13^, ^14^ Other studies that looked at the outcomes of cardiac surgery in patients with MRT in the past showed in‐hospital mortality rates ranging between 2.3% and 13.6%.^11^, ^12^, ^13^, ^14^, ^15^, ^16^, ^17^

Long‐term survival shows a variation between the different studies (all with different follow‐up periods), ranging from 32% to 80%.^11^, ^12^, ^13^, ^14^, ^15^, ^16^, ^17^ The survival at the end of this study was 72.9% at a median follow‐up of 52 months. The exact reason of this high survival is unknown. A possible explanation is the high amount of patients with breast cancer in this study. Chang et al^11^ showed in a previous study that these patients are more likely to have a positive outcome than patients with Hodgkin lymphomas. Yet, the analysis of the current study showed no significant differences in mortality between the different types of cancer. Of the 16 patients who died in this study, 10 were diagnosed with breast cancer vs 4 with Hodgkin lymphoma. Differences in the treatment regimen could explain this inconsistency. In the past, more aggressive and excessive strategies of MRT were custom.^22^ The sample of Chang et al consisted of patients who had received tangential (peripheral) radiation therapy, while the sample of this study mainly consisted of patients who underwent MRT long before surgery (mean interval of MRT‐surgery: 16 years). On top of that analyses showed no difference in outcomes between left‐sided and right‐sided breast cancer, indicating that the patients with breast cancer in this study did not receive tangential radiation therapy.

### 6.1 Limitations

As with all retrospective and observational studies, this study is subject to various limitations due to the research design. In some cases, the health records did not contain all of the desired information. A large number of patients (77.2%) were referred from other hospitals. This complicated the collecting of patient data, especially during the follow‐up period. Also, the retrospective design of this study made it impossible to collect complete data of the radiation therapy that the patients had received in the past. The gathering of data regarding the received radiation dose—which may have had a major influence on the surgery outcomes—was especially difficult. Since this was a single‐center study, only a small number of patients were eligible for inclusion. A future multicenter study should be initiated to create a larger group and draw more generalizable conclusions.

## 7 CONCLUSION

This retrospective study showed that short‐term mortality is high in patients with a history of MRT. While surgery‐related events were the main cause of short‐term mortality, malignancy‐related events were the main cause of late mortality during the follow‐up. Furthermore, this study indicated that the actual mortality in patients with a history of MRT is higher than predicted by the Euroscore II. This implicates that physicians should be careful when applying mortality risk calculators, since they tend to underestimate the mortality risk of patients with a history of MRT. Finally, a previous stroke and a lower preoperative GFR were identified as a risk factor for all‐cause mortality.

## CONFLICT OF INTERESTS

The authors declare that there are no conflict of interests.

## AUTHOR CONTRIBUTIONS

OBD contributed to data gathering, analysis, and manuscript writing; ESF contributed to manuscript co‐writing and correction; SMB contributed to echocardiography and manuscript correction; WJPB contributed to data analysis and manuscript correction; and AK contributed to patient selection and manuscript correction.

## ETHICS STATEMENT

T. Groenveld on behalf of the Medical Ethics Review Committee of the Academical Medical center:

“Referring to our letter of 20 December 2018 (reference number W18_414 # 18.479), we are pleased to confirm that the Medical Research Involving Human Subjects Act (WMO) does not apply to the above mentioned study and that an official approval of this study by our committee is not required.”

## Data Availability

The datasets used and/or analyzed during the current study are available from the corresponding author on reasonable request
